# Evolutionary and Functional Analysis of a *Chara* Plasma Membrane H^+^-ATPase

**DOI:** 10.3389/fpls.2019.01707

**Published:** 2020-01-21

**Authors:** Suyun Zhang, Myckel Habets, Holger Breuninger, Liam Dolan, Remko Offringa, Bert van Duijn

**Affiliations:** ^1^ Plant Biodynamics Laboratory, Institute of Biology Leiden, Leiden University, Leiden, Netherlands; ^2^ Plant Developmental Genetics, Institute of Biology Leiden, Leiden University, Leiden, Netherlands; ^3^ School of Food Science and Biotechnology, Zhejiang Gongshang University, Hangzhou, China; ^4^ Department of Plant Sciences, University of Oxford, Oxford, United Kingdom; ^5^ Research Department, Fytagoras BV, Leiden, Netherlands

**Keywords:** plasma membrane H^+^-ATPase, Chara, evolution, plant, algae

## Abstract

H^+^-ATPases are the main transporters in plant and fungal plasma membranes (PMs), comparable to the Na^+^/K^+^ ATPases in animal cells. At the molecular level, most studies on the PM H^+^-ATPases have been focused on land plants and fungi (yeast). The research of PM H^+^-ATPases in green algae falls far behind due to the lack of genetic information. Here we studied a potential PM H^+^-ATPase (CHA1) from *Chara australis*, a species of green algae belonging to the division Charophyta, members of which are considered to be one of the closest ancestors of land plants. The gene encodes a 107 kDa protein with all 6 P-type ATPase-specific motifs and a long, diverse C-terminal domain. A new amino acid sequence motif R*****Q in transmembrane segment 5 was identified among the known PM H^+^-ATPases from Charophyta and Chlorophyta algae, which is different from the typical PM H^+^-ATPases in yeast or land plants. Complementation analysis in yeast showed that CHA1 could successfully reach the PM, and that proton pump activity was obtained when the last 77 up to 87 amino acids of the C-terminal domain were deleted. PM localization was confirmed in Arabidopsis protoplasts; however, deletion of more than 55 amino acids at the N-terminus or more than 98 amino acids at the C-terminus resulted in failure of CHA1 to reach the PM in yeast. These results suggest that an auto-inhibition domain is located in the C-terminal domain, and that CHA1 is likely to have a different regulation mechanism compared to the yeast and land plant PM H^+^-ATPases.

## Introduction

Plasma membrane (PM) H^+^-ATPases belonging to the least divergent subfamily of the P-type ATPases, P3A, have been identified in plants, fungi, some protozoa, and archaea. Similar to their counterparts in animal cells, the Na^+^/K^+^ ATPases, PM H^+^-ATPases act as primary transporters in both plants and fungi (Kuhlbrandt, 2004; Morth et al., 2011). Protons are used to create an electrochemical gradient, to balance and regulate the cytoplasmic pH, and to facilitate secondary membrane transporters, e.g., for uptake of nutrients (Palmgren, 2001). In higher plants, the PM H^+^-ATPases are under a tight regulation of biotic and abiotic stimuli, such as plant hormones and light, and are also well known as key regulators in processes such as cell expansion, stomatal opening, and polarity development (Hohm et al., 2014).

Previous research on the PM H^+^-ATPase in plants, with regard to their protein sequence, the crystal structure, and biochemical characteristics of this highly conserved subfamily of transport proteins has shown that functional proteins have five main domains aiding different functions (Buch-Pedersen et al., 2009; Ekberg et al., 2010). There is a transmembrane domain with 10 transmembrane helices and four cytosolic domains. The cytosolic domains are defined as the N (nucleotide binding) domain, the P (phosphorylation) domain, the A (actuator) domain, and the R (regulation) domain (Pedersen et al., 2007).

In a broader evolutionary perspective, the PM H^+^-ATPases show patterns of diversity correlated to the evolutionary lines, especially in the R domain. It is reasonable to believe that these patterns may fit to the diversity of environments and are correlated to the evolution pattern. The R domain in vascular plants has been shown to function as an auto-inhibition domain, containing two conserved regions (Region I and II) and a penultimate threonine (named: pT H^+^-ATPase) (Palmgren et al., 1991; Speth et al., 1997). This threonine can be phosphorylated by protein kinases, after which it becomes a binding site for 14-3-3 proteins (Fuglsang et al., 1999). The binding of 14-3-3 proteins abolishes the (auto) inhibition and activates the pumping (Baunsgaard et al., 1998). The binding of 14-3-3 proteins can be stabilized by the addition of the fungal toxin fusicoccin to create long-lasting pump activation (Baunsgaard et al., 1998; Oecking and Hagemann, 1999; Duby and Boutry, 2009). The penultimate threonine as a key regulation point is only present in land plant PM H^+^-ATPases, and thus arose at a later time point in evolution. Experiments have shown that the moss *Physcomitrella patens* and liverwort *Marchantia polymorpha*, as the basal lineages of extant land plants, contain both PM H^+^-ATPases with and without the penultimate threonine, indicating that the pT regulation mechanism might only have developed after the transition of plants from water to land (Okumura et al., 2012a). On the other hand, no evidence of pT was found among the known PM H^+^-ATPases in lower lines such as algae (Okumura et al., 2012b; Nishiyama et al., 2018). In the PM H^+^-ATPases of Chlorophytes as well as the resembling protist, the whole R region regulation complex (Region I, II and pT) is missing, instead these PM H^+^-ATPases can have a large variety of either short or long C-terminal cytoplasmic domains (Pedersen et al., 2012).

Regarding to the main function of PM H^+^-ATPases, as a primary pump, there are several conserved essential residues creating a one-way-only passage for the transport of H^+^. For example, based on the ***A*** *rabidopsis thaliana* **H** ^+^-**A**TPase2 (AHA2) model, residue Asp684 in transmembrane segment M6 functions as the H^+^ acceptor/donor, and residue Asn106 in M2 is proposed and confirmed as a gatekeeper in cooperation with Asp684 to ensure the efficient transport of protons against the electrochemical gradient (Buch-Pedersen et al., 2000; Buch-Pedersen et al., 2003; Pedersen et al., 2007; Ekberg et al., 2013). So far, the Asp684 and Asn106 found in AHA2 are conserved among the known PM H^+^-ATPases in land plants and algae. In addition, Arg655 in AHA2 has been proposed as a backflow preventer due to its position in the cavity opposite to the Asp684, and its positive charge, which may serve as positive plug preventing proton reflux (Buch-Pedersen and Palmgren, 2003; Buch-Pedersen et al., 2009; Pedersen et al., 2012). Early evidence showed a conservation of R655 (AHA2) in all streptophyte pumps, but it is absent in typical protist and chlorophyte PM H^+^-ATPases (Pedersen et al., 2012). Coincidently, there is evidence of coexistence of both Na^+^/K^+^ and H^+^ pumps in these protists and chlorophyte algae, despite the fact that the PM Na^+^/K^+^-ATPases or PM H^+^-ATPases are strictly exclusively expressed in, respectively, animal cells or land plants/fungi (Pedersen et al., 2012). Based on this data, it was suggested that due to the lack of R655 chlorophyte proton pumps fail to build up a membrane potential, and that the primary transporter function is taken over by the coexisting Na^+^/K^+^ -ATPases (Pedersen et al., 2012). Since not enough research has been done on the function of PM H^+^-ATPases and Na^+^/K^+^-ATPases in algae, the above hypothesis is calling for further evidence.

In the evolutionary tree, there is a division of freshwater green algae, named Charophyta, members of which are considered as closest ancestors to the land plants (Zhang and van Duijn, 2014). Among the Charophyta, a group of branched, plant-like, multicellular green algae Charophyceae has already been used as a model system in plant physiology research for the past decades, credit to their huge internodal cells (Foissner and Wasteneys, 2014). For example, electrophysiological studies by measurement and control of potential difference (PD) across the plasma and tonoplast membranes of Characeae date back to the 1970s, offering the background knowledge of the plasma membrane transporters and channels including PM H^+^-ATPases (Beilby and Casanova, 2014). The Characeae show the ability of acid/alkaline band formation along the internodal cells, when stimulated by light (Fisahn and Lucas, 1995). This phenomenon is likely to be mediated by PM H^+^-ATPases, and the acid band is thought to facilitate the uptake of dissolved inorganic carbon species (Bulychev et al., 2001). As opposed to land plants, PM H^+^-ATPases in *Chara coralline* do not show obvious stimulation by the plant hormone auxin or by fusicoccin (Okumura et al., 2012b; Zhang et al., 2016), although circadian and/or seasonal variations in responsiveness cannot yet be ruled out (Coleman and Findlay, 1985; Beilby et al., 2015). This is consistent with the hypothesis that pT H^+^-ATPases did not evolve earlier than in bryophytes (Okumura et al., 2012b).

The lack of genomic sequence information on Charophyta algae has until now hampered the molecular characterization of the P3A H^+^-ATPases in this essential plant group that bridges the unicellular algae (mainly the Chlorophyta algae) and the land plants. Recently, however, an international collaborative effort has elucidated the genome and transcriptome sequences of *Chara braunii* (Nishiyama et al., 2018), and this has opened a new era of using *Chara* as a molecular research model. Based on preliminary transcriptome sequence information we identified a potential *Chara* PM H^+^-ATPases gene (*CHA1*) of *Chara australis* and analyzed the function of the predicted protein. Alignment of CHA1 with other P3A H^+^-ATPases from land plants, fungi and algae, showed both conservation and differences in the evolution pattern. Expression of CHA1 in yeast and in Arabidopsis protoplasts showed that the protein localized at the PM. Moreover, the *CHA1* gene could only complement the yeast *pma1* mutant when 77 to 87 amino acids of the C-terminus were deleted.

## Materials and Methods

### Plant Material

*C. australis* was a kind gift from Prof. Ilse Foissner in Austria and was cultured at room temperature in aquaria filled with sterilized forest soil covered with sand at the bottom and artificial pond water (APW) containing 0.1 mM KCl, 0.1 mM CaCl_2_, and 0.1 mM NaCl (pH about 6.0) as described earlier (Berecki, 2001) under 16 h photoperiod. Fresh internodal cells and branches were used for genomic DNA isolation.

### Genomic DNA Extraction and H^+^-ATPase Isolation

Chara genomic DNA was extracted from fresh Chara cells from the up-ground part (internodal cells and branches) using the CTAB DNA isolation protocol (de Pater et al., 2009).

Based on the sequences of three possible Chara PM H^+^-ATPases contigs (transcript_4956, transcript_1405, transcript_181b) obtained from a sequencing experiment on *C. australis* RNAs, the most likely open reading frames (ORFs) were identified from the three hits with CLC workbench 7, named transcript_4956 CDS (2775bp), transcript_1405 CDS (2952bp), and transcript_181b CDS (1977bp). And based on these predicted CDSs (coding sequences), the forward, reverse primers were designed to amplify the fragments from genomics DNA (respectively, 4956_F, 4956_R, 1405_F, 1405_R, 181b_F and 181b_R) (**Supplemental Table 1**). When this failed, proton pump specific forward (PPs F1) and reverse (PPs R1) primers were designed based on the most conserved part from the three hits with approximately 1 kb in between. Other forward and reverse primers were designed to cover the whole sequence with specificity based on the hit sequences (**Supplemental Table 1**). Isolated Chara genome DNA was used as template and PCR reactions were performed using the Phusion polymerase (Thermo) with GC-buffer and recommended settings; temperatures were set based on the primers or the best tested results from the gradient-temperature PCR.

Tail PCR was carried out based on the description by Liu and Whittier et al. (1995), for the extension from the isolated and sequenced middle part to both the N-terminal and C-terminal. For N-terminal extension, forward primers NT_1, NT_2, NT_3 (**Supplemental Table 1**) were used successively for the three consecutive PCR reactions, each with one of degenerative primers AD1, AD2, and AD3 (**Supplemental Table 1**), respectively. C-terminal extensions were carried out twice, stepwise, in the same way but with forward primers CT1_1, CT1_2, CT1_3 and CT2_1, CT2_2, CT2_3 (**Supplemental Table 1**).

All PCR products were purified by gel electrophoresis and recovered using a GeneJET Gel purification kit (Ehermo scientific). DNA fragments were cloned into the pJET Blunt cloning vector using the CloneJet PCR Cloning Kit (Thermo Scientific), and were subsequently sequenced (Macrogen Europe, Amsterdam, The Netherlands).

### Sequence Analysis and Gene Identification

PCR sequences were assembled and analyzed with CLC Main Workbench 7. The deduced protein sequence was analyzed by InterPro (including results from two independent tools of TMHMM server v.2.0 and Phobius) and the Protein Homology/analogy Recognition Engine Version 2 (PHYRE2).

### Yeast Strains and Culture Conditions

The yeast *S. cerevisiae* haploid null mutant strain YAK2 (Matα, ade2-101, leu2Δ1, his3-Δ200, ura3-52, trp1Δ63, lys2-801pma1-Δ:: HIS3, pma2-Δ:: TRP1) was kindly provided by Prof. Marc Boutry (University of Louvain, Belgium). This strain, lacking the two endogenous genomic copies of the PM H^+^-ATPase genes*PMA1* and *PMA2*, and carrying the *PMA1* gene under the control of the *GAL1-10* promoter on an URA3 centrometric plasmid for survival, was used for the complementation assay (de Kerchove d’Exaerde et al., 1995). The yeast *S. cerevisiae* BY4743 (MATa/α, his3Δ1/his3Δ1, leu2Δ0/leu2Δ0, LYS2/lys2Δ0, met15Δ0/MET15, ura3Δ0/ura3Δ0) was used to study the subcellular localization of CHA (Zhang, 2016).

### Plasmids Constructions

The 2µplasmid 2up(PMA1)PMA2, containing the *Nicotiana plumbaginifolia* proton pump *PMA2* gene under the control of the yeast *PMA1* promoter and the*LEU2* marker gene for selection (de Kerchove d’Exaerde et al., 1995; Luo et al.,1999), was a kind gift from Prof. Marc Boutry (University of Louvain, Belgium). The plasmid 2up(PMA1)CHA1 was derived from 2up(PMA1)PMA2 by replacing the *PMA2* gene for the *PstI-HindⅢ* fragment containing *CHA1*. For subcellular localization in yeast, the *CHA1* gene was inserted as *Spe*I-*Sal*I fragment into single-copy yeast plasmid pUG34GFP (Sakalis, 2013) for the N-terminal fusion with GFP under the control of the MET25 promoter, resulting in pUG34-GFP-CHA1. For subcellular localization in Arabidopsis protoplasts, the attB-flanked PCR fragment containing the *CHA1* gene was first inserted to pDONR207 through gateway BP recombination, and subsequently the gene was LR recombined into destination vector pART7(35S)YFP-Gateway plasmid (Gleave, 1992; Yao, unpublished data), resulting in plasmid pART7(35S)YFP-CHA1. The *CHA1* wild-type (wt) sequence and the different C-terminal (ΔC977, ΔC941, ΔC923, ΔC908, ΔC898, ΔC891, ΔC887) and N-terminal (ΔN55, ΔN64) deletion variants were obtained by PCR using pJET-CHA1 as template and the primer pairs listed in **Supplemental Table 2**.

### Complementation Assay

The yeast strain YAK2 (Matα, ade2-101, leu2Δ1, his3-Δ200, ura3-52, trp1Δ63, lys2-801pma1-Δ:: HIS3, pma2-Δ:: TRP1) was transformed with plasmid 2up(PMA1)CHA1, or with 2up(PMA1)PMA2 (de Kerchove d’Exaerde et al., 1995) as positive control (C+). An empty expression plasmid (Yeplac) was transformed as the negative control (C-). Yeast transformation was carried out with the lithium acetate method (Ito et al., 1983). The transformed yeast was plated on solid selective medium (MY medium with addition of adenine and lysine) with either 2% galactose or 2% glucose for selective expression. Independent biological repeats were carried out at least three times.

### Subcellular Localization of CHA in Yeast

The plasmids pUG34GFP and pUG34-GFP-CHA1 were transformed into yeast BY4743 cells by the lithium acetate method (Ito et al, 1983). Transformants were plated on solid MY medium containing methionine (MET) to suppress the expression of *CHA1*. After 3 days, clones were transferred to liquid MY medium containing methionine. The overnight liquid cultures were then centrifuged and the yeast cells were resuspended in fresh MY liquid medium without methionine to induce *CHA1* expression for 1 h. All yeast cultures were carried out at 30⁰C. Cells were collected by centrifugation and resuspension in MilliQ water (to lower the background noise) for the GFP signal observation. In this study, a 63x magnification oil immersion objective on the Zeiss Imager microscope (LSM510) was used. Fluorescence at 488 nm excitation and 520 nm emission was analyzed using ZEISS ZEN2009 software. The images were then processed with ImageJ (ImageJ National Institutes of Health, USA). Three independent transformations and observations were carried out.

### Arabidopsis Protoplast Transformation and Microscopic Analysis

Protoplasts were prepared from *Arabidopsis thaliana* Col-0 cell suspension cultures and transfected with the plasmid pART7-35S-YFP-CHA1 mediated by polyethylene glycol (PEG) as previously described (Schirawski et al., 2000). Transfected protoplasts were incubated at 25⁰C for at least 16 h in the dark prior to observation. The YFP signal was detected using an argon laser with 514 nm excitation and a band pass filter of 530–600 nm with a confocal imaging microscope (Zeiss LSM5 Exciter). Images were processed with ImageJ (ImageJ National Institutes of Health, USA). Three independent biological repeats were carried out to achieve a credible result.

## Results and Discussion

### Identification of a Potential PM H^+^-ATPase Gene *CHA1* in *C. Australis*

**Figure 1:**
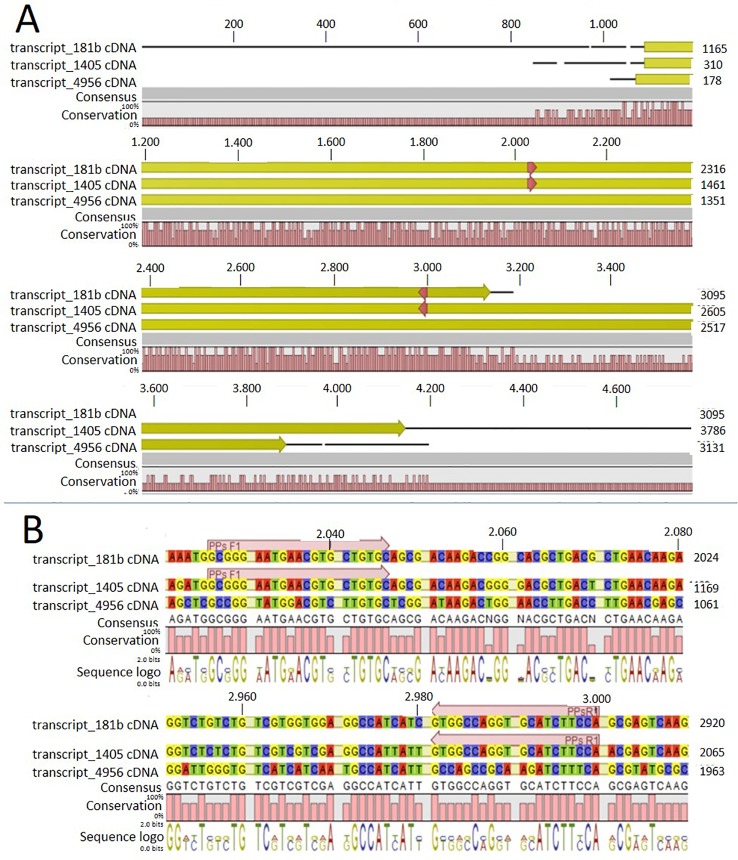
Alignment of the three contigs (transcript_181b, transcript_1405, and transcript_4956) with CLC workbench 7. Upper numbers are base pair numbers counted from the first base pair of all listed sequences. **(A)** Alignment of the contigs with the predicted ORF in yellow and red arrows indicating the primers used for amplifying the conserved part. **(B)** Sequence information in detail regarding the forward and reverse primer (PPs F1 and PPs R1).

**Figure 2:**
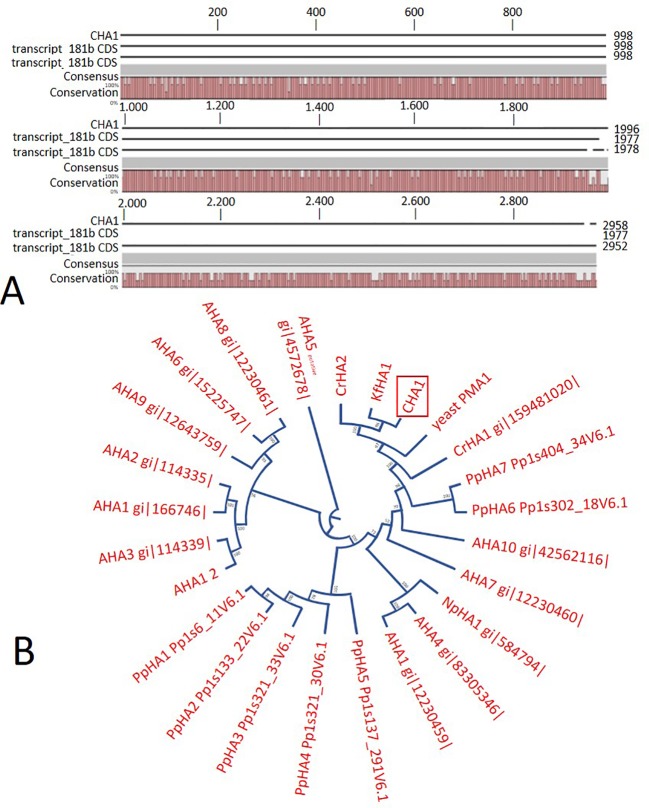
**(A)** Sequence alignment on CLC workbench 7 of the assembled full *CHA1* (from start to stop codon) with predicted coding sequences of transcript_181b and transcript_1405. Upper numbers are base pair numbers counted from the first base pair of all listed sequences. **(B)** Phylogenetic tree of P3A ATPases, with *CHA1* highlighted in a red box. AHA is short for Arabidopsis H^+^-ATPase, NpHA is referring to the plasma membrane H^+^-ATPase of *Nicotiana plumbaginifolia*, PpHA is referring to the plasma membrane H^+^-ATPase of *Physcomitrella patens*, CrHA is referring to the plasma membrane H^+^-ATPase of *Chlamydomonas reinhardtii*, and KfHA is referring to the plasma membrane H^+^-ATPase of *Klebsormidium flaccidum*.

**Figure 3:**
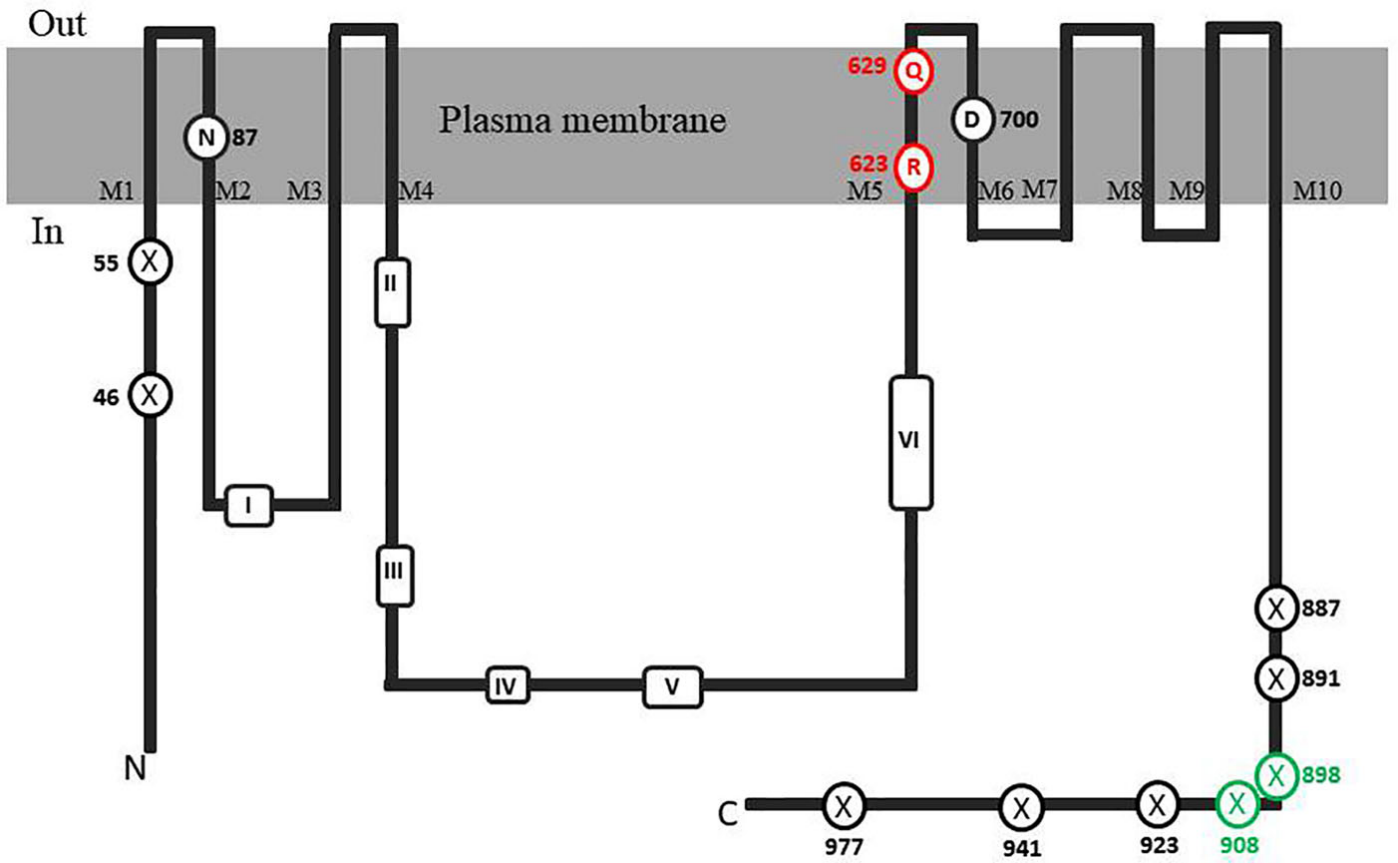
Topological model of CHA1 protein. The conserved aminol acids of PM H^+^-ATPases are circled in black. Marked conserved motifs I-VI are listed in **Table 1**. The residues marked with crossed circles indicate the truncations for activity and membrane location test. The crossed circles in green indicate the truncations that were able to rescue the null mutant yeast strain.

In order to identify possible PM H^+^-ATPase genes in *C. australis*, we searched the transcriptome database generated by high throughput sequencing on RNAs isolated from *C. australis*, using the known plant PM H^+^-ATPase protein sequences (including those of *Chlamydomonas reinhardtii*, *Physcomitrella patens*, *Nicotiana plumbaginifolia* and *Arabidopsis thaliana*) as bait. This identified three contigs named transcript_4956 CDS (2775bp), transcript_1405 CDS (2952bp), and transcript_181b CDS (1977bp). Alignment of these contigs revealed a conserved sequence of approximately 1 kb (**Figure 1A**), which we amplified from genomic DNA using primers PPs F1 and PPs R1 (detailed in **Figure 1B**). A tail PCR strategy was designed to obtain both the 5’ and 3’end sequences from the start until the stop codon with the genomic DNA. In this way, we managed to assemble a single potential PM H^+^-ATPase encoding sequence with an open reading frame length of 2,958 bp, named *CHA1* (for ***C*** *hara* ***H*** *^+^-* ***A*** *TPase*). Alignment with transcript_1405 CDS and transcript_181b CDS (**Figure 2A**) indicated that the isolated genomic sequence was not interrupted by introns. From the genomic fragment, a polypeptide with a calculated molecular mass of 107 kDa was deduced, which fits in the size range that can be found among other P3AH^+^-ATPases. A BlastP and phylogenetic analysis using the NCBI database confirmed the highest homology to the *Klebsormidium flaccidum* PM H^+^-ATPases (65% amino acid identity), and 57% or 35% amino acid identity with, respectively, the *Chlamydomonas reinhardtii* and the *Arabidopsis thaliana* PM H^+^-ATPase (**Figure 2B**). The predicted protein appeared to contain all 6 P-type H^+^-ATPase-specific amino acid motifs (**Table 1**), and a conserved aspartic acid (corresponding to Asp684 in AHA2) as the H^+^ acceptor/donor (**Figure 3**) (Serrano, 1989; Buch-Pedersen and Palmgren, 2003). During the preparation of this manuscript, the genome sequence information of *Chara braunii* was decoded by a great effort of a worldwide collaboration (Nishiyama et al., 2018). Blast analysis showed that *CHA1* closely related to *Chara braunii* P-type H^+^-ATPases *g6273* and *g30033* (Nishiyama et al., 2018).

**Table 1:** Conserved motifs of CHA1 comparing to (E-P) ATPases and their proposed functions.

Motif	Sequence	Proposed functions
I	TGES	Phosphatase activity (E)
II	DKTGTLT	Phosphorylation and transduction (D)
III	KGAP	ATP binding and/or kinase activity (K)
IV	DPPR	ATP binding (D)
V	MITGD	ATP binding (D)
VI	GDGVNDAPALK	ATP binding (D)

### CHA1 Protein Sequence Analysis

#### C-Terminal Regulation Domain

**Figure 4:**
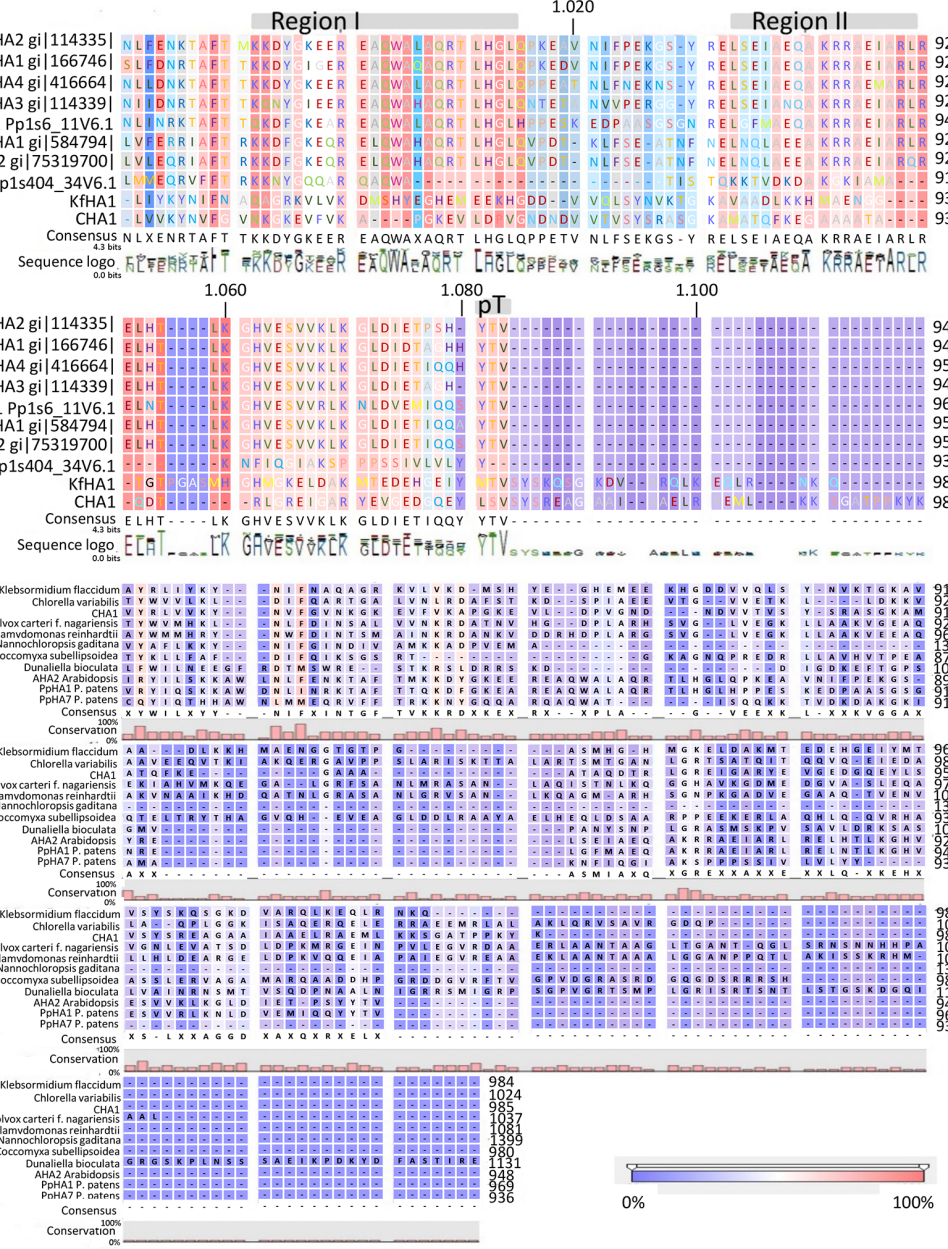
**(A)** C-terminal alignment of CHA1 with other PM H^+^-ATPases. Color bar below indicates the conservation degrees of the alignments. Gray bars above indicate the conserved C-terminal regions in land plants PM H^+^-ATPases. Upper numbers are base pair numbers counted from the first base pair of all listed sequences. **(B)** Alignment of PM H^+^-ATPases from algae and protists at the C-terminal. Color bar below indicates the conservation degrees of the alignments. Upper numbers are base pair numbers counted from the first base pair of all listed sequences.

Analysis of the C terminal (R-domain) showed that the well-conserved region I and II and the penultimate threonine (pT) in land plant PM H^+^-ATPases are missing in CHA1 (**Figure 4A**). This is consistent with the suggestion by Okumura and his coworkers that pT PM H^+^-ATPase most likely only appeared in bryophytes (Okumura et al., 2012b), as expected from the P3A H^+^-ATPases evolution perspective (Pedersen et al., 2012). Alignment of the C-terminal domain with that of P3AH^+^-ATPases from the Chlorophyta, Charophyta and some protists showed little homology, and no clear domain pattern could be identified (**Figure 4B**). Considering the diversity of the living environment of the algae and protists (fresh water/salinity water, with/without light, etc.) the PM H^+^-ATPase would be regulated under different conditions such as difference in salt tolerance, by different regulators for the different species and circumstances (Beilby and Casanova, 2014). Further analysis by, e.g., mutation studies should be carried out to understand the regulation mechanism of H^+^-ATPases in different *Chara* species and other algae, and by relating this to their habitats it might shed new light onto the role of PM H^+^-ATPases in plant tolerance to environmental conditions.

#### Up-Hill Transport Capacity.

**Figure 5:**
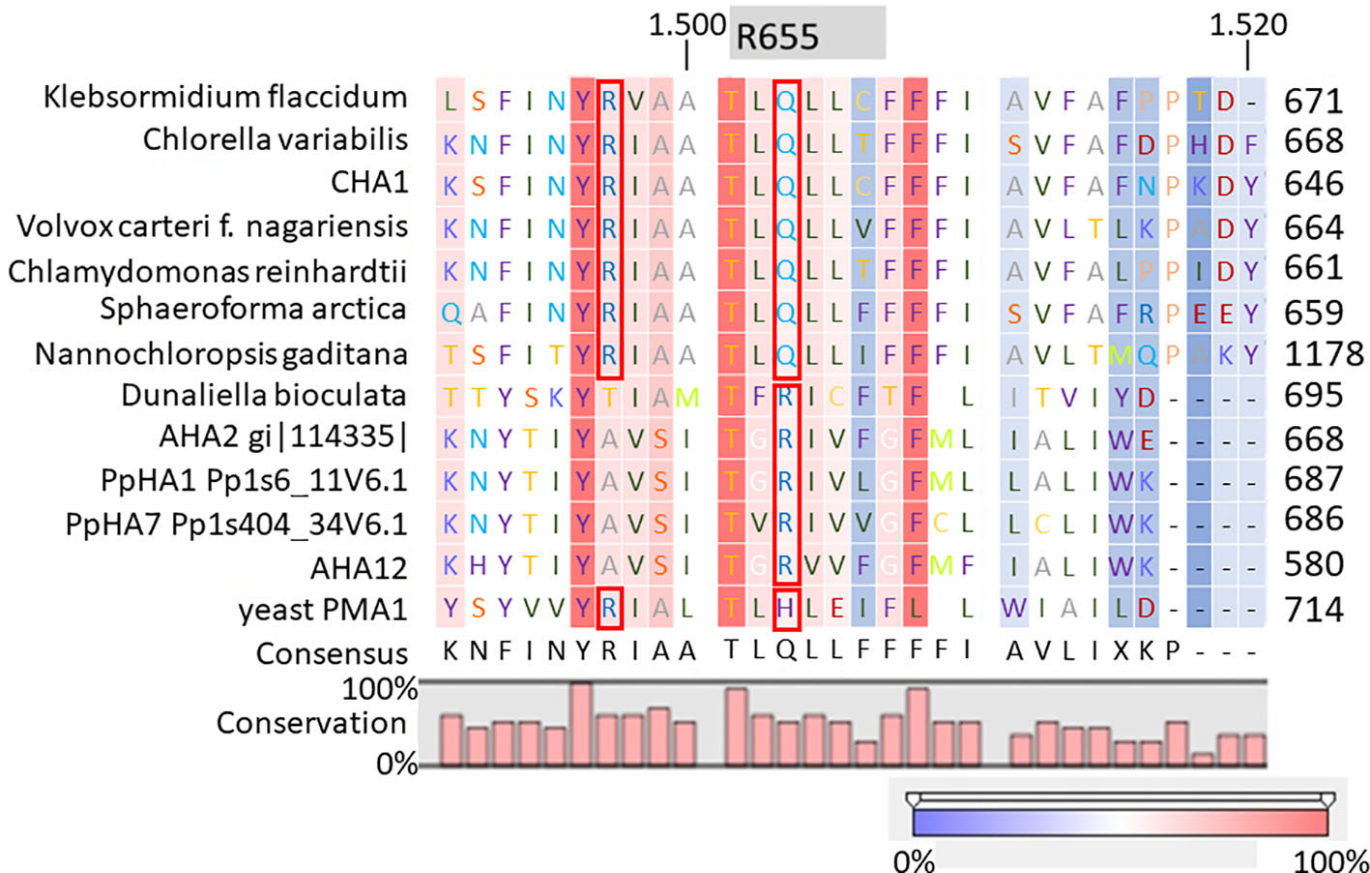
Alignment of transmembrane segment M5 (based on AHA2) of CHA1 and other P3A H^+^-ATPases. Gray boxes indicate the residue and number in AHA2. Red boxes indicate certain conserved residues which may evolve with the proton up-hill transport ability. Color bar below indicates the conservation degrees of the alignments. Upper numbers are base pair numbers counted from the first base pair of all listed sequences.

It is well-established that PM H^+^-ATPases are able to build a high electrical membrane potential difference (PD) across the fungal and plant plasma membranes up to -300 and -200 mV, respectively. In the steady state, at neutral pH and in light, with the proton pump in control, certain Characean algae (*Chara, Nitella*) cells could also reach a membrane potential of -200 mV or even lower (Lucas, 1982; Beilby and Casanova, 2014). From these values, it seems that the PM H^+^-ATPases of these algae also have a strong capacity to pump the protons in the up-hill direction, building up a huge chemical-electrical gradient. The alignment of amino acid sequences of CHA1 and other known sequences ofP3A H^+^-ATPases from algae, yeast, and plants shows an asparagine in position 87 of CHA1 corresponding to the gate-keeper residue Asn106 in the transmembrane segment M2 (**Figure 3**). Interestingly, the “positive plug” Arg655 in M5 is neutralized by a hydrophilic glutamine in CHA1 (Gln629), and this glutamine seems to be the dominant residue among the other algal species at this position (**Figures 3** and **5**). Instead, an arginine shows up at six residues in front of the same transmembrane segment M5, as Arg623 in *Chara*, which seems also quite conserved among the PM H^+^-ATPases of the algal species. At the same position in yeast *Saccharomyces cerevisiae* PMA1, there is also an arginine (Arg695). Early research found that in yeast this positive charged Arg695 in M5 together with the negative charged Asp730 in M6, formed a salt bridge linking M5 and M6, turning out to be important for the structure stability of PMA1 (Gupta et al., 1998). The positively charged His701 in yeast PMA1, which aligns with the positively charged Arg655 in AtAHA2 (**Figure 5**), also has an essential role in the protein folding and location functioning, which is dominant lethal when mutated (Dutra et al., 1998). This fact makes it difficult to check whether the His701 (PMA1) would have the “plug effect” in yeast, resulting a higher PD (-300 mV). Thus, in certain number of algal PM H^+^-ATPases, a positive charged residue in the middle of M5 is missing, comparing to the ones in yeast and land plants. However, there is a quite conserved arginine at six residues in front, similar as in yeast PMA1. The question would be whether this arginine in algae functions as a “salt bridge” to maintain the protein structure or as a “positive plug” to support the up-hill proton transport. The well-studied electro-physiology properties of *Chara* combining with mutation studies of CHA1 might give some structural and functional hints to this new pattern of R*****Q in M5 among these algae species.

### Complementation Analysis of *CHA1* in Yeast

**Figure 6:**
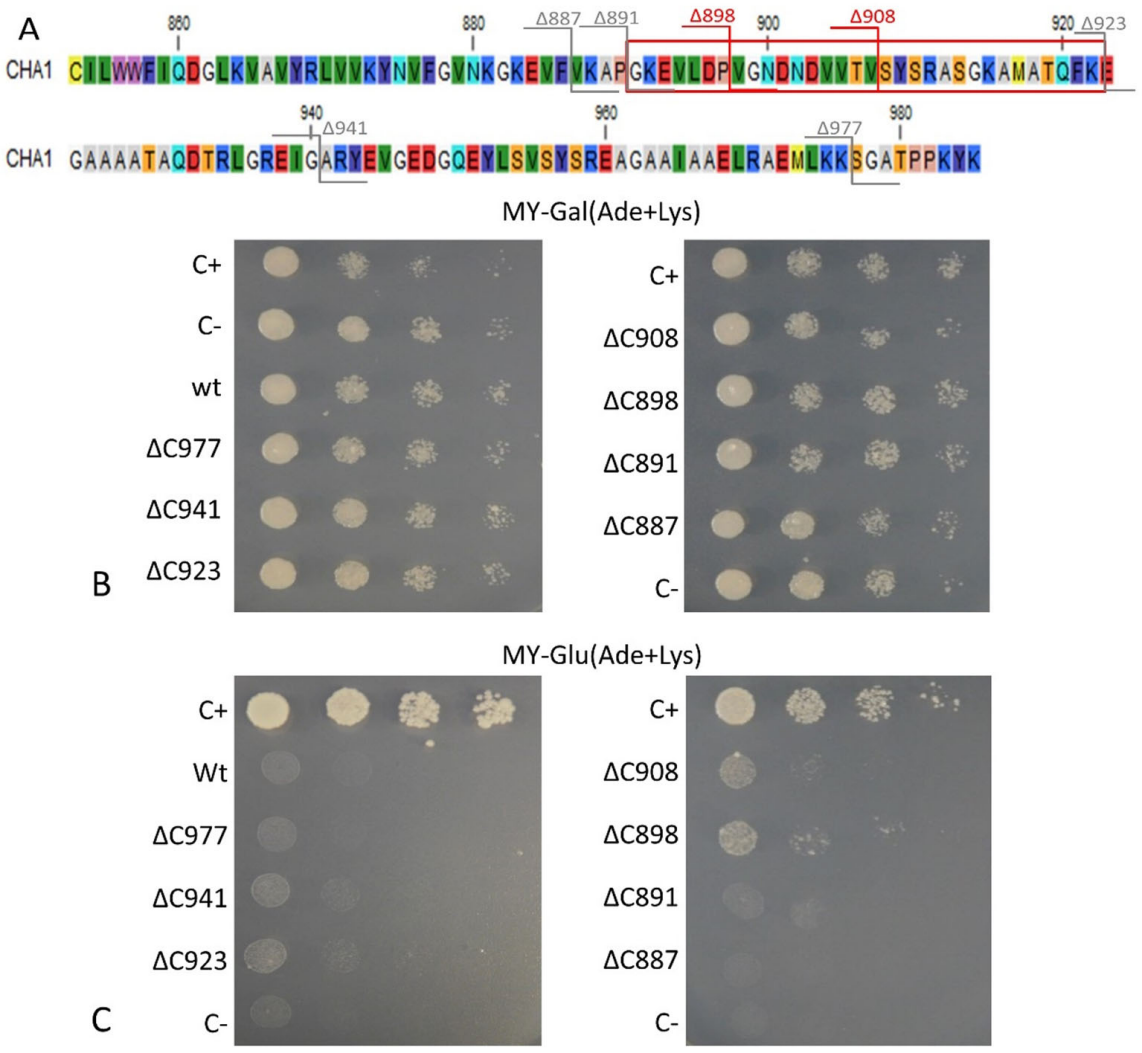
**(A)** C-terminus of CHA1. Red numbers and cutting lines indicate the positions in CHA1 from where C-terminal deletions were made that rescue the null mutant yeast strain. Gray numbers and cutting lines indicate the truncation sites of CHA1 that failed to rescue the null-mutant yeast strain. The red box indicates the potential regulation domain. Different residues are marked with different background colors. **(B, C)** Complementation assay of wild-type and 7 C-terminally truncated versions of CHA1 (ΔC977, ΔC941, ΔC923, ΔC908, ΔC898, ΔC891, ΔC887). Transformants were serially 10-fold diluted (as shown from left to right as dilutions series) and dropped onto solid selection medium (MY medium addition with adenine, lysine) with either galactose (Gal) or glucose (Glu). One representative result out of four independent biological repeats is shown here. **(B)** Yeast YAK2 strains grow on Gal-medium expressing both yeast PMA1 (under the control of Gal1 promotor) and heterologous CHA1 (under the control of yeast PMA1 promotor, indicated wt), C-terminal truncated genes (under the control of yeast PMA1 promotor, indicated ΔC977, ΔC941, ΔC923, ΔC908, ΔC898, ΔC891, and ΔC887, respectively) together with positive (C+) and negative (C-) control. **(C)** Same yeast strains as in B (indicated wt, ΔC977, ΔC941, ΔC923, ΔC908, ΔC898, ΔC891, and ΔC887, respectively) grown on Glu-medium without galactose), only expressing heterologous CHA1 and truncated versions under control of the yeast PMA1 promotor. Positive (C+) and negative (C-) control are included as well.

To confirm that the isolated *CHA1* gene functions as a proton pump and to further investigate the function of the C-terminal as an auto-inhibition domain, the full length CHA1 gene (985 amino acid, indicated as wt in the figures) and the C-terminal step-wise deletion mutants ΔC977, ΔC941, ΔC923, ΔC908, ΔC898, ΔC891, or ΔC887 (**Figure 6A**), were sub-cloned into the yeast expression plasmid and transformed into the yeast YAK2 null mutant strain.

Results showed that on galactose medium, yeast YAK2 strains could survive well when both the *PMA1* and *CHA1* genes were expressed (**Figure 6B**). When culturing on glucose medium, the expression of only CHA was not sufficient to support the yeast growth (**Figure 6C**). As (parts of) the C-terminus may act as an auto-inhibitory domain, the absence of survival of the yeast YAK2 strain with only a complete CHA1 expressed may be due to auto-inhibitory-induced inactivity of the H^+^-ATPase. To verify this hypothesis, seven different lengths of C-terminal truncations were also tested under the same condition (ΔC977, ΔC941, ΔC923, ΔC908, ΔC898, ΔC891, ΔC887). Two tested mutants (ΔC908 and ΔC898) showed the capacity to support the growth of yeast, though at a lower level as compared to the *Nicotiana plumbaginifoliaPMA2* gene (C+) (**Figure 6C**). The truncation of the last 87 amino acids (ΔC898) conferred the highest rescue ability to the *CHA1* gene (**Figure 6C**).

Interestingly, the *CHA1* complementation results in yeast are similar to what has been observed for *AHA2* (Palmgren and Christensen, 1993). Also here a C-terminal deletion was required to boost the activity of the pump and thus to partially support the growth of the null mutant yeast (**Figure 6**). In conclusion, CHA1 can function as a proton pump in yeast provided that part of the C-terminus is removed. This suggests that, like in AHA2, the C-terminal domain of CHA1 (in particular, between Gly891 and Glu923) harbors a regulatory (auto-inhibition) domain. However, an alignment of the C-terminal domain of CHA1 with that of PM ATPases of fungi and higher plants did not identify conserved motifs (**Figure 4B**). Also, the plant H^+^-ATPases classical 14-3-3 binding motif could not be detected in CHA1. This opens to question how CHA1 is regulated by the C-terminal domain, which remains an interesting target for further studies.

### CHA1 Localization in Yeast (N-Terminal GFP Fusion)

When Arabidopsis AHA1-3 were expressed in yeast, they did not complement the pma1 mutant, as some failed to enter the secretory pathway to be properly targeted to the PM. This seemed to be the most likely cause of unsuccessful complementation (Villalba et al., 1992; Palmgren and Christensen, 1993). Since a similar situation may be true for CHA1, we investigated this possibility by expressing an N-terminal GFP-CHA1 fusion in the yeast system.

**Figure 7:**
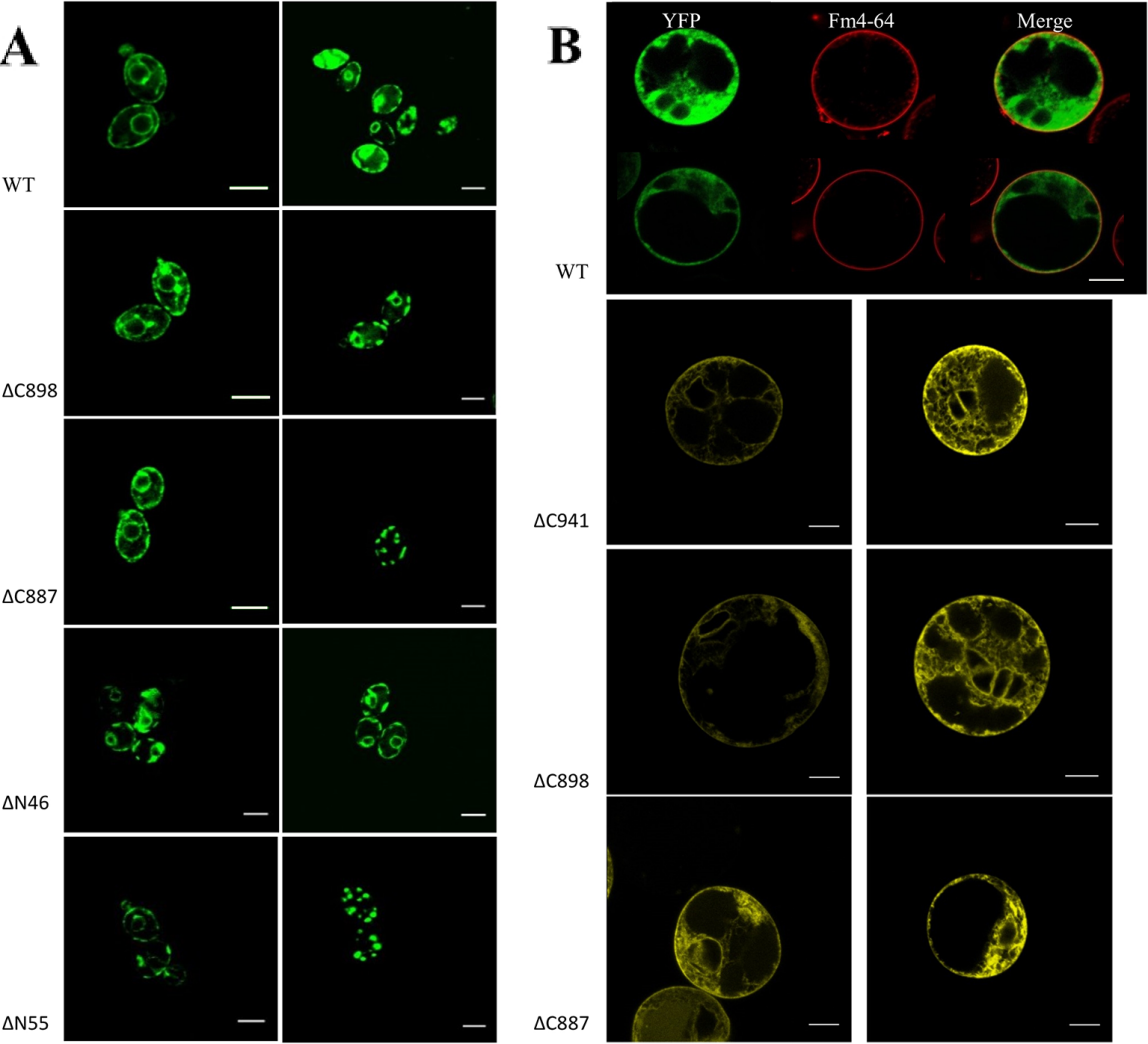
**(A)** Subcellular localization of N-terminal GFP-CHA1 fusion protein and its N/C-terminal truncated versions in yeast BY4743 strains. Two identical localization samples of each line are presented from three independent transformations. Wild-type YFP-CHA1 shows uneven PM and perinuclear localization. The C-terminal truncated version ΔC898-CHA1 also shows uneven-PM and perinuclear localization. C-terminal truncated version ΔC8887-CHA1 shows either uneven-PM, perinuclear localization or a punctate cytoplasmic-pattern. N-terminal truncation of ΔN46-CHA1 shows the same uneven-PM and perinuclear localization. N-terminal truncation of ΔN55-CHA1 shows a weak PM signal in a small portion of cells, while most of the cells show punctate cytoplasmic pattern. Scale bars, 5 µm. **(B)** Localization of N-terminal YFP-CHA1 fusion and C-terminally truncated mutant versions in *Arabidopsis thaliana* Col-0 protoplasts, two representative results out of three independent biological repeats are shown. WT: Cells expressing N-terminal YFP fusion with wild type CHA1 in green (artificial color, indicated YFP), Fm4-64 plasma membrane dye in red (indicated Fm4-64), and the merged picture of both (indicated Merge). ΔC941: cells expressing N-terminal YFP fusion with C-terminal truncated CHA1-ΔC941, with both PM and ER localization. ΔC898: cells expressing N-terminal YFP fusion with C-terminal truncated CHA1-ΔC898, with both PM and ER localization. ΔC887: cells expressing N-terminal YFP fusion with C-terminal truncated CHA1-ΔC887, with both PM and ER localization. Scale bars: 10 µm.

Based on the analysis of three independent transformations, we conclude that GFP-CHA1 expressed in the yeast BY4743 shows strong signals on plasma membrane and on some cytoplasmic membrane systems. In contrast to the visible continuous circle observed for the PM-localized yeast PMA1 (Mason et al., 2006), GFP-CHA1 showed a punctured circle representing PM-localized fusion protein, and a strong perinuclear signal, most likely representing ER-localized fusion protein (**Figure 7A**), consistent with the Arabidopsis AHA2 expression in yeast (Regenberg et al., 1995). The GFP-CHA1 versions with C-terminal deletions ΔC898 (which can support the growth of yeast) showed the same localizations as the wild type CHA1, whereas the non-rescue version ΔC887 showed the PM signal in a few cells, while most cells showed a speckled cytoplasmic GFP signal.

We further tested the N-terminal truncations of CHA1 based on the predicted transmembrane segments by online software PHYRE2 and TMHMM server v.2.0, truncation of the first 46 amino acid at N-terminal cytoplasmic domain (ΔN46), and truncation of the first 55 amino acid (ΔN55) including the whole N-terminal cytoplasmic domain and part of first transmembrane helix (M1). ΔN46 showed the same pattern as the wild type CHA1 gene, while further truncation (incomplete M1) clearly has a negative influence on the protein targeting or stability; thus, a cytoplasmic staining pattern as with ΔC887 could also be seen with ΔN55 (**Figure 7A**).

In conclusion, CHA1 can be synthesized and is likely transported to the yeast plasma membrane, but it is not able to support the yeast growth. With a C-terminal deletion of 87 amino acids, CHA1ΔC898 is transported to the PM and is also sufficiently active in the yeast cells to partially compensate for the loss of the yeast proton pumps. This indicates that the C-terminal domain of CHA1 functions as an (auto)inhibitor of the pump activity, at least in the yeast system, and this inhibition cannot be eliminated by the yeast regulation system. Biochemical experiments are necessary to identify the enzymatic properties of the CHA1 with/without C-terminal truncations, such as the transport kinetics, pH profile, and regulation mechanisms. Also, it seems that the integrity of the first transmembrane helix and ~20–30 amino acids after the last transmembrane helix are essential for the proper targeting and stability of the protein. Any sabotage may cause it to become trapped in cytoplasmic bodies (Mason et al, 2006; Mason et al., 2014).

### CHA1 Localization in Plant Protoplast (N-Terminal Fusion With YFP)

To study the expression and sub-cellular localization of CHA1 in plants, an N-terminal YFP-CHA1 fusion was expressed in *Arabidopsis thaliana* Col-0 protoplasts from the viral *35S* promoter (*p35S::YFP-CHA1*). In three independent transformation experiments YFP-CHA1 showed strong cytoplasmic localization, with a relatively weaker signal on the plasma membrane (**Figure 7B**). Mutant fusion proteins with a truncated C-terminus (ΔC887, ΔC898, ΔC941) showed the same localization as the wild-type –YFP-CHA1 protein.

Even though the plasma membrane localization signals were not so stable or strong in the protoplasts, they indicated that at least part of the produced YFP-CHA1 protein is correctly transported and inserted into the PM. The reason for the strong internal signal is unclear, but it suggests that either biosynthetic secretion is inefficient, or that the fusion protein is readily internalized.

## Summary

A potential PMH^+^-ATPase (CHA1) was isolated from *C. australis*. Sequence analysis indicated it as a P3A ATPase, with the diversity in the regulation domain and the proton transport cavity, which showed a new perspective in the PMH^+^-ATPases evolution pattern. Functional and mutational studies need to be carried out to confirm its biogenesis characters.

The functionality of the Characeae proton pump is known from the generation of large negative transmembrane potential differences of up to -250 mV (Beilby and Casanova, 2014). By complementation in yeast we could show that the isolated CHA1 has proton pump functionality, as it can partially rescue the proton pump-lacking yeast strain. This activity could only be found in a C-terminal truncated version of the protein, although both the wild-type and the truncated versions are targeted to the plasma membrane. This was shown in an assay using N-terminal GFP fusion to the protein. This suggests that CHA1 regulation is prone to a C-terminal localized auto-inhibitory process, which can be circumvented by deletion. Further truncation of C-terminal disrupts the plasma membrane localizing, thus results in that way in a non-functional pump. Also, in Arabidopsis protoplasts the wild-type CHA1 protein and the C-terminal deleted proteins are expressed in the plasma membrane. However, the details of the regulation mechanism of CHA1 still remain unclear. More biochemical experiments with the wild type, single point mutations, and N/C-terminal truncations should be carried out *in vitro* to profile the enzymatic properties of the pump. Expressing fluorescently labelled CHA1 in *Chara* internodal cells may also facilitate the functional research of the sophisticated Chara membrane structures such as charaosomes in the mechanism of the pH banding pattern in *Chara* species (Beilby and Casanova, 2014).

## Data Availability Statement

The raw data supporting the conclusions of this article will be made available by the authors, without undue reservation, to any qualified researcher.

## Author Contributions

SZ and BD contributed conception and design of the study. SZ performed the experimental work. HB and LD provided the sequences of three possible *Chara* PM H^+^-ATPases contigs. SZ and MH performed the bio-informatic analysis. SZ wrote the manuscript. RO and BD supervised the project. All authors contributed to manuscript revision and approved the submitted version.

## Funding

This work was supported by the China Scholarship Council (2011635073) and Single Cell Research Foundation (SSCR06-01-15).

## Conflict of Interest

The authors declare that the research was conducted in the absence of any commercial or financial relationships that could be construed as a potential conflict of interest.
