# Supplementary material for: Evolutionary and Functional Analysis of a Chara Plasma Membrane H+-ATPase
Source: Front Plant Sci. 2020 Jan 21;10:1707. doi: 10.3389/fpls.2019.01707 (PMC6985207; doi:10.3389/fpls.2019.01707)
Supplement: Supplementary file 1 [file DataSheet_1.pdf]

Supplemental materials

Supplemental table 1.

Primers used for gene isolation

Primer name	5'-3' DNA Sequence
1405_F0	ATGGGGCAGGAGGAAGAGCGTAAGGGC
1405_R0	TCACTTGTGCTTGCGCGGCGGCGGC
4956_F0	ATGCCTCTCAAGAGTAACGGCG
4956_R0	TTACTTGGCTCGCGTATCCATCGAGCG
181b_F0	ATGGGGCACGAAGACGGGGGTAAGGG
181b_R0	TCACTTCTTCTTCGCCTGGTAGAGCGC
PPs F1	GCGGGAATGAACGTGCTGTGC
PPs R1	TGGAAGATGCACCTGGCCAC
181b_CT1-T1	GACTTCCTGACTCTCTTC
181b_CT1-T2	CGATGGGTTGGTTCTGGA
181b_CT1-T3	CTCATCTCTCTCGGCATCT
181b_CT2-T1	GACTTCCTGACTCTCTTC
181b_CT2-T2	CGATGGGTTGGTTCTGGA
181b_CT2-T3	CTCATCTCTCTCGGCATCT
181b_NT-T1	CCTTGCCATCCCTCTTTA
181b_NT-T2	GCATTACCTGCCTTCGTC
181b_NT-T3	GATAAGGATGGGCATAGG
AD1	NTCGASTWTSGWGTT
AD2	NGTCGASWGANAWGAA
AD3	WGTGNAGWANCANAGA
181_b F1	CGACCTCTGCGAGAACAAGCGG
181_b F2	CAAGGAGAGGTGGAGGCGACC
181_b F3	GGCAAGGTGCAGACCATCG
181_b F4	GAAGGTGACCCCCAGTTGG
181_b F5	GGGATCCACCTGCGTGCAAGG
181_b F6	GGATGCTCGGTGGGTATGACCGG
181_b R2	CTGGATAACCATCTTGTTCAGCG
181_b RP3	GAGATGCCGAGAGAGATGA
181_b CTR	CTACTTGTACTTGGGCGGCGTT

Supplemental table 2.

Primers used for plasmids construction

Primer name	5’-3’ DNA Sequence	Purpose
GF_CHA	GGGGACAAGTTTGTACAAAAAAGCAGGCT TCATGGGGCAGGATGAGGGGGGTA	pART7(35S)YFP-CHA
GR_CHA	GGGGACCACTTTGTACAAGAAAGCTGGGT CCTACTTGTACTTGGGCGGCGT	
GR_ ΔC887	GGGGACCACTTTGTACAAGAAAGCTGGGT CCTAGAACACCTCCTTGCCCTT	
GR_ ΔC898	GGGGACCACTTTGTACAAGAAAGCTGGGT CCTATGGGTCAAGCACCTCCTT	
GR_ ΔC941	GGGGACCACTTTGTACAAGAAAGCTGGGT CCTAGCCGATTTCCCTTCCCAG	
CHA_F_SpeI	GACTAGTATGGGGCAGGATGAGGGGGGTA AG	pUG34-GFP-CHA
ΔN46_F_SpeI	GACTAGTATGGTCATCTTCTTGCGGCAG	
ΔN55_F_SpeI	GACTAGTATGCCTATGCCCATCCTTATCT	
CHA_R_SalI	GCGTCGACCTACTTGTACTTGGGCGGCGTT GCG	
ΔC887_R_SalI	GCGTCGACGAACACCTCCTTGCCCTT	
ΔC898_R_SalI	GCGTCGACTGGGTCAAGCACCTCCTT	
CHA_F_PstI	GCTGCAGATGGGGCAGGATGAGGGGGGTA AG	2μp(PMA1)CHA
CHA_R_HindIII	CCAAGCTTCTACTTGTACTTGGGCGGCGT	
ΔC887_R_HindIII	CCAAGCTTCTAGAACACCTCCTTGCCCTT	
ΔC891_R_SalI	GCGTCGACCTAAGGAGCCTTCACGAACAC	
ΔC898_R_HindIII	CCAAGCTTCTATGGGTCAAGCACCTCCTT	
ΔC908_R_SalI	GCGTCGACCTAGACGGTCACCACATCGTT	
ΔC923_R_SalI	GCGTCGACCTACTTGAACTGCGTAGCCAT	
ΔC941_R_HindIII	CCAAGCTTCTAGCCGATTTCCCTTCCCAG	
ΔC977_R_ SalI	GCGTCGACCTACTTCTTCAACATCTCGGCC CT	
